# Case Report: Anesthetic Management of Cesarean Section in a Patient With Paraplegia

**DOI:** 10.3389/fmed.2022.783796

**Published:** 2022-05-11

**Authors:** Yongchun Su, Xiaofeng Lei, Jin Yu

**Affiliations:** ^1^Department of Pediatrics, Chongqing YouYouBaoBei Women and Children's Hospital, Chonqing, China; ^2^Department of Anesthesiology, Chongqing Health Center for Women and Children, Chonqing, China

**Keywords:** anesthesia, cesarean section, paraplegia, autonomic hyperreflexia, spinal cord injury

## Abstract

**Background:**

With the advancement of medical science and rehabilitative care, more women with spinal cord injury (SCI) can conceive. However, autonomic hyperreflexia due to SCI complicates anesthesia management during cesarean sections.

**Case Presentation:**

This study reports the anesthesia management in a woman with paraplegia with a T6 SCI lesion who underwent a cesarean section. It also reviews the anesthesia strategies used in other studies. Spinal anesthesia with a low concentration of ropivacaine was administered along with dexmedetomidine for sedation. Stable hemodynamics were achieved without complications.

**Conclusions:**

Based on the reported case and literature review, we conclude that the intrathecal block is the preferred choice for women with paraplegia who require cesarean section if the lumbar bone structure allows puncture attempts.

## Introduction

The global incidence of spinal cord injury (SCI), both traumatic and non-traumatic, is approximately 40–80 cases/million (1). Pregnancy is rare in patients with SCI. However, in recent times, pregnancies in patients with paraplegia have a good prognosis because of advanced care and better knowledge. Yet, more complications, such as autonomic hyperreflexia (AHR), may occur during labor and delivery due to the pathophysiological changes induced by SCI. In women with paraplegia, AHR is the most serious complication, and 85% of mothers with paraplegia may develop AHR if the level of SCI exceeds T6; AHR manifests as sudden and severe hypertension or even cardiovascular and cerebrovascular incidents.

There are very few reports on anesthesia management during cesarean section in patients with SCI. Studies documenting perioperative anesthetic management of pregnant women with SCI are equally rare. In this study, we report our experience of intraoperative anesthesia management in a parturient with paraplegia with SCI above T6 using spinal anesthesia with a low concentration of ropivacaine combined with dexmedetomidine for sedation. This case study has been approved by the Ethics Committee of the Chongqing Health Center for Women and Children, and informed consent was obtained.

## Case Description

The patient was a 29-year-old woman, G_1_ P_0_, at 37+6 weeks of gestation. She had a history of traumatic thoracolumbar and cervical injuries (cervical compression fractures and thoracic burst fractures) that had occurred in 2009; she had received a blood transfusion of ~4,000 ml at that time. The current level of paraplegia was T6, and the sensory and motor abilities of the limbs below the xiphoid process were completely lost. The patient complained of occasional flushing, fever, and sweating above the xiphoid process. In 2019, she decided to undergo *in-vitro* fertilization with the implantation of two frozen embryos, and one had survived. She visited the anesthesia clinic 1 month before the due date. She was diagnosed with Hashimoto's thyroiditis in 2003 and consumed levothyroxine sodium tablets during pregnancy for hypothyroidism. Thyroid function was checked regularly with recent administration of levothyroxine 100 μg/day. Diagnoses at admission were pregnancy with hypothyroidism, 37+6 weeks of gestation, G_1_ P_0_ waiting for delivery, high paraplegia, and embryo transplantation.

## Anesthetic Management

Because of the high paraplegia and the condition of the pelvis, a cesarean section was scheduled at 38 weeks of gestation. Preoperative physical examination revealed a pregnant woman (height, 160 cm; and weight, 64 kg) with light peripheral edema, Mallampati class 2 airway with a 6-cm thyromental distance, and a total absence of sensation below T6, including spinal cord reflexes and temperature sensation. Her vital signs were as follows: body temperature, 37°C; pulse, 87 bpm; respiration rate, 20 breaths/min; and blood pressure, 129/71 mm Hg. Preoperative laboratory examinations, including routine blood and urine tests, liver and kidney function, electrolytes, coagulation function, immunity, and thyroid function, showed no obvious abnormalities. There were no abnormalities on the ultrasound of the chest or lower limbs.

Electrocardiogram, pulse rate, oxygen saturation, and non-invasive cuff blood pressure were closely monitored in the operating room. Venous access to the left and right forearms was established. A radial artery puncture was also performed on the right side to monitor the internal arterial blood pressure. Baseline vital signs were as follows: heart rate, 78 bpm, SpO_2_, 99%; blood pressure, 148/75 mm Hg, and respiration rate, 21 breaths/min. Fetal heart rate was 140–150 bpm with minimal long-term variability. The patient was placed in the right knee-chest position. After successfully completing the L3/4 epidural puncture, a 25G lumbar puncture needle was inserted through the opening of the epidural puncture needle, guided by an 18G needle. A total of 3 ml of 0.3125% ropivacaine (heavy specific gravity, 0.75% ropivacaine 1.25 ml + 5% glucose 1.75 ml) was administrated into the subarachnoid space after the arachnoid membrane was punctured. Considering the possibility of epidural adhesion caused by a previous surgery, no epidural catheter was placed before the withdrawal of the lumbar puncture needle. The level of the sensory block did not exceed T4.

The neonate (3,050 g) was removed 5 min later, and the Apgar score was 10–10–10 at 1–5–10 min. The parturient was followed up with dexmedetomidine (initial dose 0.5 ug/kg/10 min, maintenance dose 0.3 ug/kg/h) intravenously. No obvious hemodynamic response was observed during the procedure. Her blood pressure remained within 15% of her baseline pressure (148/75 mmHg), and her heart rate fluctuated between 60 and 80 bpm throughout. No other analgesia was administered after the operation and during the postoperative period between surgery and discharge. The maternal vital signs were stable, and there were no complaints of discomfort except for slight pain in the surgical area.

## Discussion

With socioeconomic development and individual preference, more women with paraplegia would want to become pregnant. For delivery, the cesarean section is suitable for some women with paraplegia considering specific conditions, although paraplegia itself is not a contraindication for vaginal delivery (2). Another factor and indication to perform an elective cesarean section is the patient's inability to feel labor pains (1). A retrospective cohort study of 15 patients with paraplegia reported a cesarean section rate of 47% (indications were obstetric reasons) (2).

The pathophysiological changes of SCI make anesthesia management more complicated during cesarean sections in a parturient with paraplegia, and AHR triggered by noxious and distension stimuli below the level of the injury during childbirth is the most severe life-threatening complication (3). As a result of uterine contractions, any parturient with SCI whose injury is at T6 or above is at a risk of acute AHR, which is usually mistaken for preeclampsia at onset (4).

In women with paraplegia, AHR may occur during cesarean sections either by general or regional anesthesia, and analgesic insufficiency is a strong inducing factor in AHR (5). The impact of introducing various methods of anesthesia on patients with paraplegia and SCI has been investigated in previous studies, including the general anesthesia (6–8), epidural anesthesia (4, 9), spinal anesthesia (1, 5, 10), transversus abdominis plane block (11), and even no anesthesia (12).

Cross et al. (12) reported that the cesarean section was performed in 43% of 22 SCI patients with 30 deliveries. Among the 13 deliveries that involved cesarean section, epidural anesthesia was used in seven patients, general anesthesia in five patients, and no anesthesia in one patient. Fantin et al. (11) reported the case of a patient with high thoracic flaccid paraplegia (T3/4 lesion) who underwent elective cesarean delivery by ultrasound-guided lateral TAP blocks; the authors believe that these blocks lowered the risk of AHR during surgical incision.

In fact, epidural or spinal anesthesia is recommended as an intrathecal block for parturients with paraplegia undergoing cesarean delivery (7). Spinal anesthesia, which prevents AHR better by achieving a more predictable neural blockade at the T4 level, is preferred.

The optimal level of sensory block for cesarean section is T4. The patient presented in this study had a cervical and thoracic spine fracture due to trauma and had a history of surgery. A part of the lumbar vertebral gap was still available for induction of spinal anesthesia. Therefore, spinal anesthesia with low-concentration analgesia (0.3125% gravity ropivacaine) was used, and the epidural catheter was not placed because of possible postoperative adhesions (from previous surgeries) in the epidural space. However, it is also necessary to prepare for the possibility of spinal anesthesia not reaching the expected level of efficacy (13).

Preoperative anxiety is more severe in parturients with paraplegia than in ordinary parturients. Women with SCI who decide to get pregnant need special healthcare services (14). Therefore, auxiliary sedation is necessary to relieve parturient tension and anxiety during surgery if spinal anesthesia is selected. Excellent sedation was achieved in the reported case using a dexmedetomidine intravenous pump after the fetus was delivered. The stability of vital signs, blood pressure, and heart rate of the mother was maintained in this case, and no complications occurred during the entire anesthesia process.

In conclusion, spinal anesthesia, which likely prevents AHR better, is preferred for cesarean delivery in parturients with paraplegia in cases that allow puncture attempts. Preoperative evaluation and pre-arranged plans are necessary and important to prevent related complications as AHR. Dexmedetomidine can achieve the desired sedation effect in conscious SCI parturients selected for neuraxial anesthesia.

## Data Availability Statement

The original contributions presented in the study are included in the article/supplementary material, further inquiries can be directed to the corresponding author/s.

## Ethics Statement

The studies involving human participants were reviewed and approved by Ethics Committee of Chongqing Health Center for Women and Children. The patients/participants provided their written informed consent to participate in this study. Written informed consent was obtained from the individual(s) for the publication of any potentially identifiable images or data included in this article.

## Author Contributions

YS collected the patient's information and wrote the manuscript. XL and JY performed the anesthesiology procedures. All authors approved the final manuscript.

## Funding

This study was supported by the Natural Science Foundation Project of Chongqing, Chongqing Science and Technology Commission (No. cstc2021jcyj-msxmX0763).

## Conflict of Interest

The authors declare that the research was conducted in the absence of any commercial or financial relationships that could be construed as a potential conflict of interest.

## Publisher's Note

All claims expressed in this article are solely those of the authors and do not necessarily represent those of their affiliated organizations, or those of the publisher, the editors and the reviewers. Any product that may be evaluated in this article, or claim that may be made by its manufacturer, is not guaranteed or endorsed by the publisher.
